# Alpha-2-macroglobulin from circulating exosome-like vesicles is increased in women with preterm pregnancies

**DOI:** 10.1038/s41598-020-73772-z

**Published:** 2020-10-12

**Authors:** Júlia A. Tronco, Bruna R. de A. Ramos, Natália M. Bastos, Sérgio A. Alcântara, Juliano C. da Silveira, Márcia G. da Silva

**Affiliations:** 1 Department of Pathology, Botucatu Medical School, São Paulo State University (UNESP), Botucatu, SP Brazil; 2 Department of Veterinary Medicine, Faculty of Animal Science and Food Engineering, São Paulo University (USP), Pirassununga, SP Brazil; 3 Department of Mophology, Biosciences Institute, São Paulo State University (UNESP), Botucatu, SP Brazil

**Keywords:** Molecular biology, Diseases

## Abstract

Preterm labor (PTL) and Preterm Premature Rupture of Membranes (PPROM) impose substantial morbimortality on mothers and newborns. Exosomes act in intercellular communication carrying molecules involved in physiopathological processes. Little is known about exosomal proteins in prematurity. Our aim was to evaluate the protein expression of hemopexin, C1 inhibitor (C1INH) and alpha-2-macroglobulin (A2M) from circulating exosomes of women with PTL and PPROM. Plasma was obtained from PTL, PPROM, Term in labor and Term out of labor (T) patients, exosomes were isolated by ultracentrifugation, then lysed and the proteins quantified. Western Blot (WB) and Nanoparticle Tracking Analysis (NTA) were performed. Data were compared by Kruskal–Wallis, unpaired T-test and one-way ANOVA. WB and NTA confirmed exosome isolation (concentration: 4.3 × 10^10^ particles/ml ± 1.9 × 10^10^). There was no difference regarding hemopexin or C1INH expression between the groups. For A2M, the fold change was significantly higher on preterm groups when compared to term groups (1.07 ± 0.30 vs. 0.42 ± 0.17, *p* < 0.0001). Higher levels of A2M in circulating exosomes are linked to preterm pregnancies. sEV are strong candidates to intermediate maternal–fetal communication, carrying preterm labor-related immunomodulatory proteins.

## Introduction

Preterm labor (PTL) is defined by labor before the 37th week of gestation and affects about 10% of all pregnancies, representing a global public health challenge^1–3^. Preterm birth, a consequence of this condition, is the main cause of neonatal and infant morbidity and mortality. Another obstetric complication associated with PTL that can aggravate maternal and fetal morbimortality is Preterm Premature Rupture of Membranes (PPROM). PPROM is characterized by the rupture of fetal membranes before 37 weeks of pregnancy and can be identified in 30–40% of PTL cases^4^.

Molecularly, mechanisms of inflammation, oxidative stress and autophagy are known to be directly involved in PTL and PPROM pathogeny^5–7^. Nevertheless, there are still no reliable biomarkers capable of predicting such conditions. This difficulty is due to the complexity that involves the interactions between maternal and fetal compartments and the difficulty to obtain good markers from relatively not invasive biological samples.

Promisingly in this field, extracellular vesicles from maternal circulation, especially small extracellular vesicles enriched in exosomes (sEV), may enclose potential biomarkers for gestational complications. Exosomes have a circled and flatted shape, are composed of a lipid bi-layer^8^ and their diameter varies between 30 and 150 nm^9^. Earlier described as a cellular elimination mechanism, the exosomes have started to stand out as a finely regulated mechanism of intercellular communication^9^. Furthermore, one outstanding characteristic of such vesicles is their high stability, as they maintain their integrity even after thawing^10^.

Recent reports show the importance of these vesicles in intercellular communication also during the gestational period. The trophoblastic and placental cells secrete sEV and it has already been demonstrated that its concentration enhances significantly over the gestational trimesters and is significantly higher on pregnant women when compared to non-pregnant individuals^11^.

sEV carry many molecules such as nuclear, cytosolic and membrane proteins, lipids, and RNAs. Some of the proteins already described to be contained in exosomes are the tetraspanins (global markers), like CD63, CD81 and CD9, EGFR proteins and major histocompatibility complex MHC I and II (originated from antigen-presenting cells), as well as other transmembrane proteins (LAMP1, TFR).

On the prematurity context, little is known about the relevance of microvesicle’s proteins and the development of PTL and PPROM. Menon et al.^12^ recently established the protein content of maternal plasma exosomes and demonstrated a correlation between the protein’s pathway and preterm and term birth’s mechanism. A report by Cantonwine et al.^13^ demonstrated that out of 132 protein evaluated from circulating microvesicles from women on the first trimester of pregnancy, 20 were related to term labor as protection factors or were risk factors for preterm labor. Three of these proteins were already described to be enclosed by exosomes (ExoCarta), stand out for their potential correlation with prematurity: hemopexin, C1 inhibitor (C1INH), and alpha-2-macroglobulin (A2M).

Considering the importance of sEV to pregnancy along with their characteristics of specificity, stability, and accessibility, it becomes evident the need to investigate molecules contained in these vesicles in women with unfavorable pregnancy outcomes, once these molecules have the potential to predict pathologies in the future and to be used therapeutically. We hypothesize that the proteins hemopexin, C1INH and A2M are differently expressed in circulating sEV from women on the third trimester with PTL or PPROM.

## Results

The sociodemographic data of the patients enrolled in the study are presented in Table 1. As expected, the mean gestational age was significantly higher for the term groups when compared to the preterm groups (275.0 ± 7.4 vs 239.1 ± 23, *p* < 0.0001). There was a significant difference also in the way of delivery and marital status between the groups. Vaginal delivery represented 71.4% of PTL, 66.6% of PPROM and 50% of TL, all term women out of labor underwent caesarian section. As for marital status, 89% of PTL, 19% of PPROM and 100% of TL and T reported being in a civil union. There was no significant difference between the groups regarding the variables: maternal age, BMI, ethnicity, parity rate, smoking, years of study, previous history of PTL or PPROM and previous abortion (Table 1).

**Table 1:** Sociodemographic data from patients included in the study.

Variables	PTL (n = 9)	PPROM (n = 7)	TL (n = 8)	Term (n = 10)	*p*
Maternal age (years)*	25.7 ± 4.0	26 9 ± 7.0	23.1 ± 4.8	29.5 ± 4.7	NS
GA at delivery (days)*	244.8 ± 20^a^	231.7 ± 26^a^	279 ± 9^b^	272 ± 3.7^b^	< 0.0001
BMI (kg/h^2^)*	26.6 ± 3.5	29.1 ± 6.7	26.9 ± 2.8	28.9 ± 4.8	NS
**Delivery (%)**					
Vaginal	71.4 (5/7)	66.6 (4/6)	50 (4/8)	–	0.0096
Caesarean	28.6 (2/7)	33.4 (2/6)	50 (4/8)	100 (10/10)	
**Marital status (%)**					
Single	11 (1/9)	71 (5/7)	–	–	0.0004
Civil union	89 (7/9)	29 (2/7)	100 (7/7)	100 (9/9)	
**Self-reported ethnicity (%)**					
White	78 (7/9)	57 (4/7)	14 (1/7)	60 (6/10)	NS
Non White	22 (2/9)	43 (3/7)	86 (6/7)	40 (4/10)	
**Parturity (%)**					
First pregnancy	33 (3/9)	43 (3/7)	71 (5/7)	40 (4/10)	NS
Multiple pregnancy	67 (6/9)	57 (4/7)	29 (2/7)	60 (6/10)	
**Smoking (%)**					
Smoking	11 (1/9)	14 (1/7)	–	10 (1/10)	NS
Non smoking	89 (8/9)	86 (6/7)	100 (8/8)	90 (9/10)	
**Years of study (%)**					
< 8 years	–	14 (1/7)	–	11 (1/9)	NS
≥ 8 years	100 (8/8)	86 (6/7)	100 (7/7)	89 (8/9)	
**Previous history of PTL/PPROM (%)**					
Presence	33 (3/9)	14 (1/7)	–	11 (1/9)	
Absence	67 (6/9)	86 (6/7)	100 (8/8)	89 (9/9)	NS
**Prior abortion (%)**					
Presence	33 (3/9)	14 (1/7)	–	20 (2/10)	NS
Absence	67 (6/9)	86 (6/7)	100 (7/7)	80 (8/10)	

The letters ‘a’ and ‘b’ represent statistical differences.

*PTL* preterm labor, *PPROM* Preterm Premature Rupture of Membranes, *TL* term in labor, *GA* gestational age, *BMI* body mass index, *NS* non-significant (*p* > 0.05).

*Data represented by mean ± standard deviation and compared by ANOVA. Qualitative variables were analyzed by Chi-square.

Regarding neonatal data, weight at birth was significantly higher among the term groups when compared to preterm groups and there was no significant difference between groups for Apgar score 10 and newborn gender (Table 2).

**Table 2:** Newborn clinical data of the patients included in the study.

Variables	PTL (n = 9)	PPROM (n = 7)	TL (n = 8)	Term (n = 10)	*p*
Weight (g)*	2362 ± 630.4^a^	2266 ± 419.5^a^	3240 ± 421.1^b^	3319 ± 460.0^b^	0.0003
Apgar 10*	9.3 ± 0.8	8.8 ± 0.8	9.7 ± 0.5	9.7 ± 0.5	NS
**Sex (%)**					
Female	33.3 (3/9)	28.6 (2/7)	50 (4/8)	60 (6/10)	NS
Male	66.7 (6/9)	71.4 (5/7)	50 (4/8)	40 (4/10)	

The letters ‘a’ and ‘b’ represent statistical differences.

*PTL* preterm labor, *PPROM* Preterm Premature Rupture of Membranes, *TL* term in labor, *NS* non-significant (*p* > 0.05).

*Data represented by mean ± standard deviation and compared by ANOVA.

**Figure 1:**
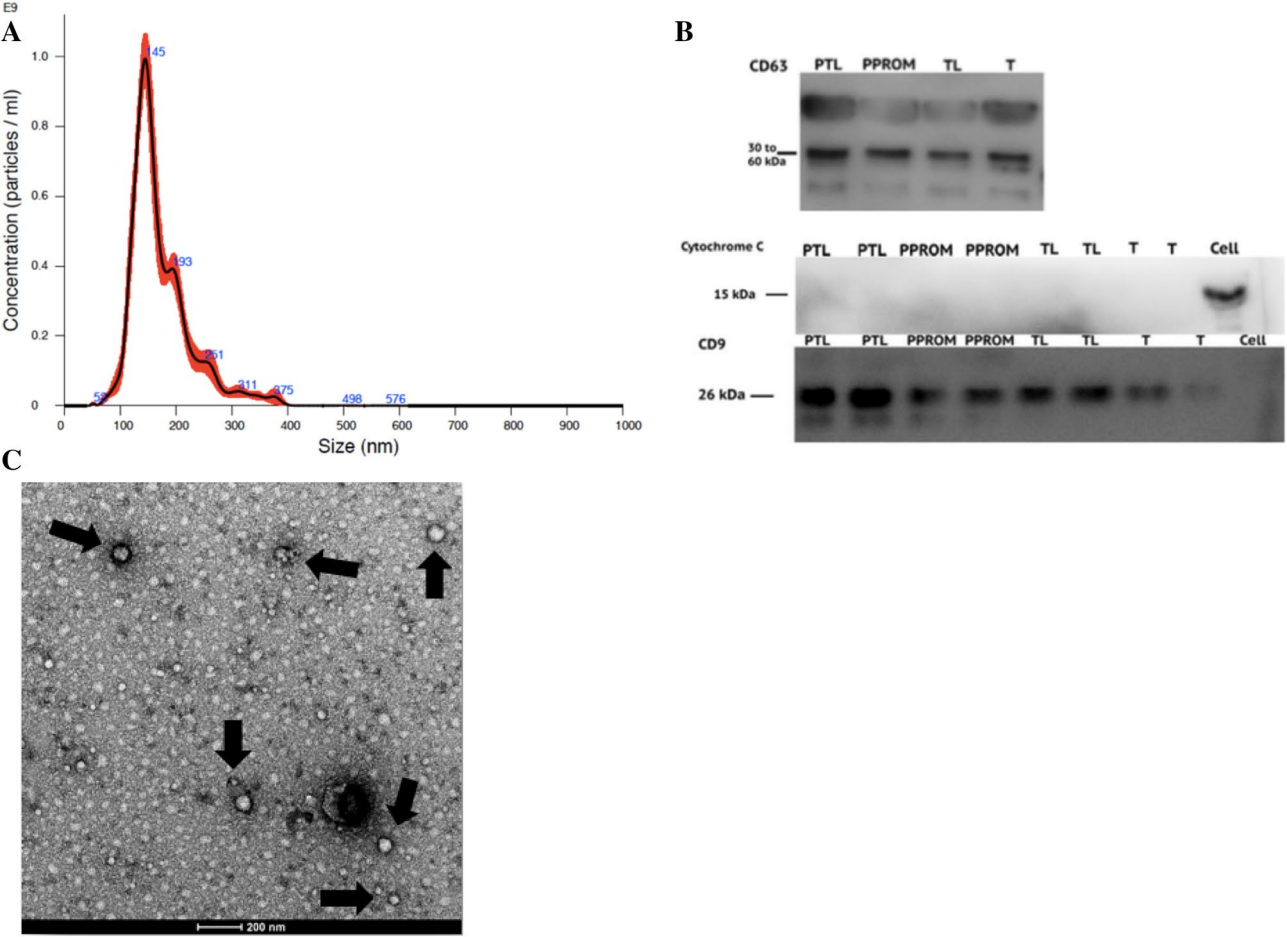
Characterization of exosome isolated from plasma. (**A**) The size distribution of sEV isolated from plasma. (**B**) Representative Western Blot for exosome marker CD63 and CD9 and negative marker Cytochrome C. (**C**) Transmission Electron Microscopy confirming the presence of sEV and its morphology characterization.

Successful sEV isolation was confirmed by Nanoparticle Tracking Analysis and Western Blot. The overall mean of particle’s mode size was 148.0 nm ± 27.5, a size compatible with sEV characterization, and the mean particle’s concentration was 4.3 × 10^10^ particles/ml ± 1.9 × 10^10^. There was no statistical difference in size or concentration of total circulating sEV across the groups. The positive controls—CD63 and CD9—were identified and the negative control—cytochrome C—was confirmed to be absent by WB (Fig. 1). Data regarding protein quantification and NTA for each group are displayed in Table 3.

**Table 3:** Comparison of protein quantification and NTA data.

Group	μg/ml	No. particles/ml	Mean (nm)	Mode (nm)
PTL	874.2	4.15 × 10^10^	178.1	150.9
PPROM	716.0	4.60 × 10^10^	173.9	148.0
TL	955.3	4.64 × 10^10^	180.8	152.8
T	656.3	4.12 × 10^10^	173.2	143.5

Mean protein quantification using Pierce BCA Protein Assay and mean values of the parameters measured by NTA for each group.

**Figure 2:**
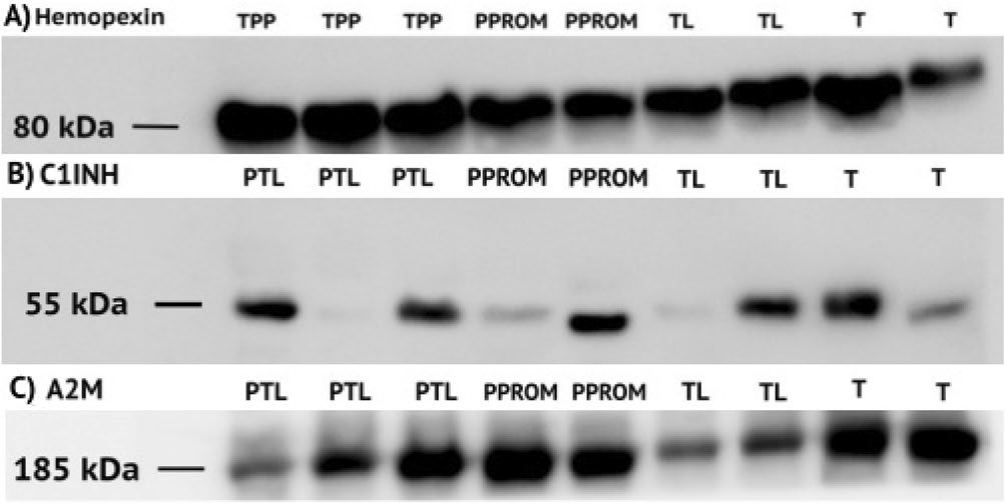
Western Blot for proteins of interest. (**A**) Representative WB for Hemopexin with 80 kDa. (**B**) Representative WB for C1INH with 55 kDa. (**C**) Representative WB for alpha-2-macroglobulin (A2M) with 185 kDa.

**Figure 3:**
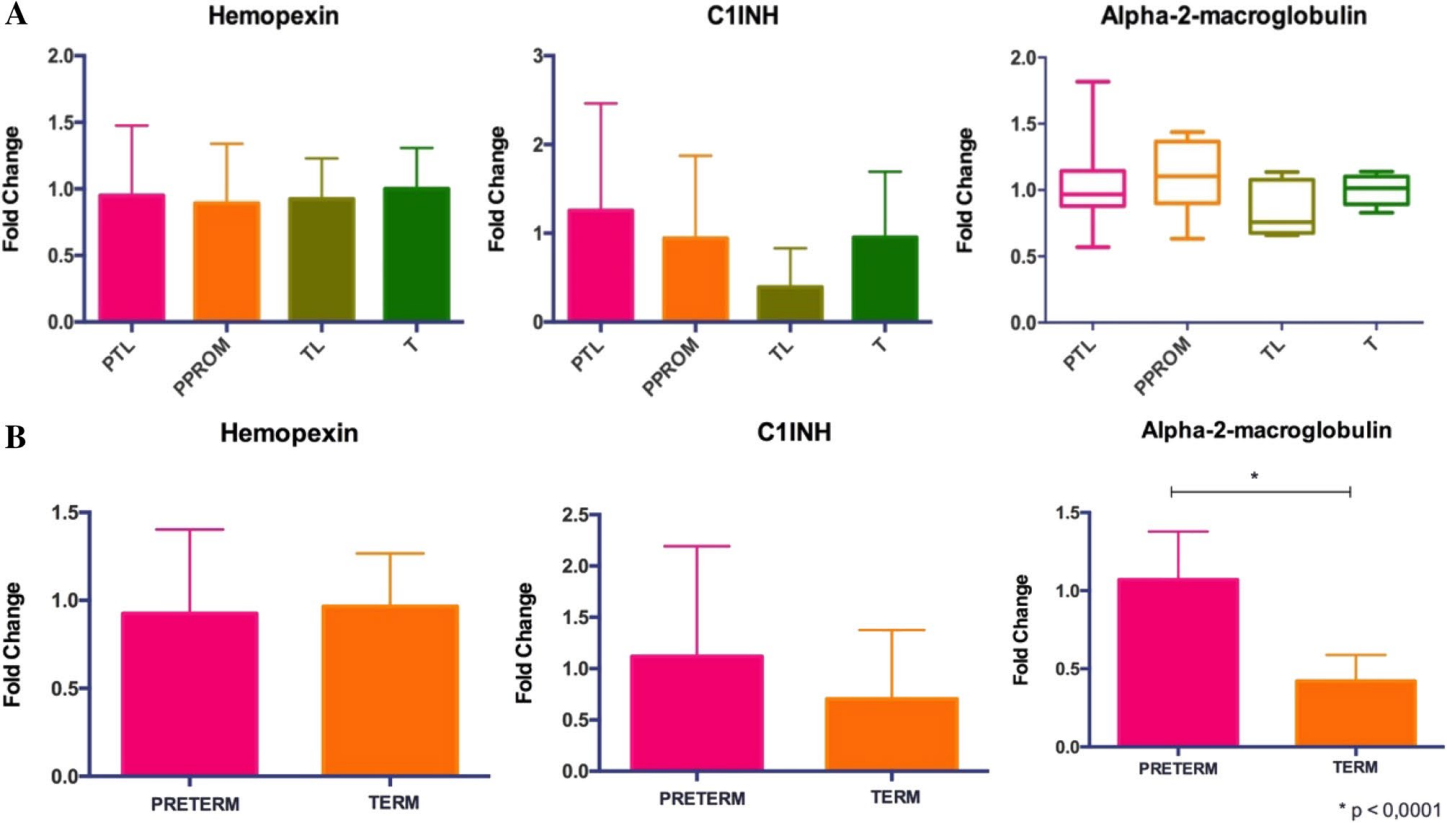
Expression of each protein of interest measured by fold change. (**A**) Hemopexin and C1INH were analyzed by One-Way ANOVA (bar graphs representation) and A2M by Kruskal–Wallis (box-whiskers representation). No significant difference was found [*p* < 0.05]. (**B**) Preterm Groups (PTL + PPROM) and Term Groups (TL + T) were analyzed by t-test. There was a significant difference in the expression of alpha-2-macroglobulin, the protein was higher expressed on the Preterm Group when compared to the Term group (*p* < 0.001).

To analyze the sEV proteins from Western Blot gels (Fig. 2), the fold change comparison for each protein was performed between the four groups and no significant difference was observed between any of them for the three proteins of interest (Fig. 3A). Further analysis included the fold change comparison between Preterm groups (PTL + PPROM) and Term groups (TL + T) (Fig. 3B). In this comparison, alfa-2-macroglobulin expression was significantly higher on preterm pregnancies compared to term (1.07 ± 0.30 vs 0.42 ± 0.17, *p* < 0.0001).

## Methods

### Patients

Blood samples were collected at the Clinics Hospital from Botucatu Medical School and at the city’s Basic Health Units, SP—Brazil, from January 2017 to August 2019. This research was approved by the Ethics Research Committee of our Institution (CAAE: 61138116.8.0000.5411). We confirm that this research was performed following relevant guidelines and regulations. Patients were included in four different groups: Preterm Labor (PTL), Preterm Premature Rupture of Membranes (PPROM), term in labor (TL), and term out of labor (T). Patients were fasting for one hour and signed the research informed consent form. Exclusion criteria included major comorbidities such as diabetes mellitus, pre-eclampsia, arrhythmia, chorioamnionitis, placenta previa, or intake of anti-inflammatory drugs and insomnia.

### Blood collection

Blood samples were collected from patients eligible for each group at the moment of their admission to the Obstetrics Services—PTL (n = 9), PPROM (n = 7), TL (n = 8), and T (n = 10)—in sterile EDTA tubes. Blood collection followed the standard time-period prior defined for all samples (always between 9:30 a.m. and 10:30 a.m.) to standardize the results and the first 1–2 ml of blood was discarded, following the recommendation from the International Society for Extracellular Vesicles^14^. Plasma was obtained by centrifugation at 1800*g* for 10 min and stored at − 80 °C until further analysis.

### Isolation of sEV

sEV’s were isolated using aliquots of 1 ml of plasma following the ultracentrifugation protocol adapted from Lässer et al^15^. First, samples were centrifuged at 20,000*g* for 30 min to remove cell debris*.* Then, the supernatant was transferred to a falcon tube and diluted with 2 ml of cold sterile phosphate-buffered saline (PBS). This mix was filtered using a sterile syringe filter with pore size 0.20 µm and transferred to the ultracentrifugation tubes which, after balanced, were centrifuged at 120,000*g* for 90 min. At this stage, the supernatant was discarded and the pellet containing the sEV was resuspended in 200 µl of cold sterile PBS and stored in sterile tubes at − 80 °C. All the centrifugation stages were performed at 4 °C.

### Nanoparticle Tracking Analysis

All samples were analyzed by Nanoparticle Tracking Analysis (NanoSight300). Samples were diluted in a 1:200 ratio and the parameters used for analysis were temperature 38.5 °C, 30 s capture, and 5 readings. Mode and concentration for each sample were given by the NanoSight software. Besides characterization, we hypothesized that the preterm birth (PTL and PPROM) could influence the sEV’s size and concentration. To confirm or refute this hypothesis, both variables for each sample were analyzed by one way-ANOVA test and t-test.

### Transmission Electron Microscopy

Transmission Electron Microscopy was performed to visually confirm sEV’s isolation and to characterize its morphology.

### Protein extraction and quantification

The buffer extraction containing Tris–HCl, NaCl, Triton ×-100, CaCl and protease inhibitor was added (50 μl) to 100 μl of exosomes resuspended in PBS, to lysate and extract the proteins. The isolated proteins were quantified by the Pierce BCA Assay kit (Thermo Scientific).

### Western Blot

Western Blot was performed to identify two positives and one negative marker to confirm successful sEV isolation. As exosome’s surface markers, CD9 and CD63 were used as positive controls and the mitochondrial marker Cytochrome C as a negative control. This technique was also performed to identify and quantify the proteins hemopexin, C1INH and A2M (Santa Cruz Biotechnology, Inc.) using the second anti-body m-IgGκ BP-HRP (Santa Cruz Biotechnology, Inc.). After protein quantification, 10 μl of Mercaptoethanol and Laemmli Buffer 4 × (1:10 ratio) were added to each sample (5 μg of protein). The tubes containing samples and buffer (approximately 20 μl total volume) were heated to 95 °C on thermocycler for 5 min to denature the proteins. At this stage, molecular weight protein standard (Precision Plus Protein Dual Color Standards by Bio-Rad) and samples were loaded into the polyacrylamide gel. The technique’s standards steps were followed and the nitrocellulose membranes were incubated with primary antibody overnight at 4 °C and then with secondary antibody for one hour and thirty minutes. The equipment *ImageQuant 4000* was used for detection.

### Image J and statistical analysis

The results obtained from Western Blot were analyzed by *ImageJ.* Normalization was performed in two steps. First, we normalized each sample’s staining band area by its respective Ponceaus’s staining band area (reference in Supplementary Material). Then, we normalized each result by the reference control samples’ results (term group). We included at least two samples of each group for every gel. By this analys is a fold change for each protein of interest was provided. The data obtained was used to perform Kruskal–Wallis and One-Way ANOVA to compare the four groups and t-test to compare two groups (Preterm Groups vs. Term groups, PTL vs. TL or PPROM vs. T) on *Prism 6* and statistical significance adopted was 5%.

## Discussion

Despite the scientific efforts to fully elucidate preterm birth markers and mediators, there are still many challenges to be overcome in the field. The investigation of specific proteins in circulating sEV is a new and promising approach. In this study, we were able to demonstrate higher expression of alpha-2-macroglobulin in preterm groups when compared to term groups. However, no significant difference regarding the expression of Hemopexin or C1INH was found between groups.

Hemopexin is a heme scavenger protein and has a complementary role to bind extracellular heme that is released as a metabolite when hemoglobin is degraded. It is mainly synthesized in the liver^16^. The usual concentration of hemopexin in plasma is 0.5–1 mg/ml. However, this concentration can increase during inflammatory events, since hemopexin is an acute-phase protein, and its production is known to be regulated by the cytokines interleukin (IL)-6, IL-11, IL-1β, leukemia inhibitory factor, oncostatin M, and tumor necrosis factor (TNF)-α^17^. As inflammatory syndromes, we expected to find higher levels of this protein on preterm samples, but there was no difference in hemopexin expression in the present study. Thus, it is worth to investigate other acute-phase proteins in the setting of PTL and PPROM.

The protein C1INH belongs to the SERPIN family and is responsible for inhibiting the spontaneous activation of C1 on complement activation. Consequently, it avoids overproduction of anaphylatoxins that act as inflammation mediators. According to Regal et al.^18^, deficiencies in specific complement regulators during pregnancy may predispose to excess complement activation and, conversely, several pregnancy complications including preterm birth have already been associated with excessive or misdirected complement activation^18^. Nevertheless, the relationship between the complement system and preterm labor is still controversial and poorly understood. While one study recently reported an association of this protein with PTL on 1st trimester samples but in an unexpected direction^13^, in our study C1INH was not differently expressed in third trimester preterm samples.

Alfa-2-macroglobulin functions as a broad-spectrum proteinase inhibitor. This protein is responsible for enhancing some procoagulant properties^19^. Besides, A2M can also bind to several cytokines, such as interleukin-6 (IL-6), Platelet-derived Growth Factor (PDGF), TNF-α and IL-1β. Corroborating with Cantowine et al.^13^ that recently demonstrated an association between this protein and PTL as a risk factor, the protein A2M was significantly higher on preterm groups when compared to the term groups in our study. Also, according to Shimomura et al.^19^, A2M might play an important role in the interactions between several cytokines and the process of inflammation. Still, little is known about this protein in the prematurity context and even less regarding sEV. Future studies in distinct populations may further investigate the role of A2M as a potential biomarker on first trimester samples.

The main limitation of this study is that the technique used does not allow us to completely exclude the possibility of microvesicles contamination (particles larger than 200 nm) or platelet derived-EV contamination (during the freeze–thaw process). While there are still no perfect isolation protocols for EVs, we were able to follow most of ISEV recommendations for sEV isolation and the possible presence of other particles does not undermine the value of this paper, as α2M could be a biomarker of preterm birth regardless of particle size. Moreover, the technique used to quantify protein’s expression, Western Blot, allows only a semi-quantitative analysis. Other techniques, such as liquid chromatography and mass spectrometry (LC/MS) could expand the knowledge in this field in future studies.

As preterm birth is still to be fully elucidated, the main strength of this study is that this is the first on our population to assess such conditions evaluating exosomal protein content. Follow up studies on this subject comparing total plasma proteins and EV proteins cargo may allow us to delve into the complex role of these vesicles in maternal–fetal communication.

We conclude that sEV are strong candidates to intermediate maternal and fetal communication, carrying immunomodulatory proteins, such as A2M, that can be related to preterm labor. Nevertheless, more studies that thoroughly investigate such proteins in the context of spontaneous prematurity are needed to extend this knowledge.

## Supplementary information

Supplementary Figures.
