# Supplementary material for: Alpha-2-macroglobulin from circulating exosome-like vesicles is increased in women with preterm pregnancies
Source: Sci Rep. 2020 Oct 12;10:16961. doi: 10.1038/s41598-020-73772-z (PMC7552414; doi:10.1038/s41598-020-73772-z)
Supplement: Supplementary file 1 — Supplementary Figures. [file 41598_2020_73772_MOESM1_ESM.docx]

**Alpha-2-macroglobulin from circulating exosome-like vesicles is increased in women with preterm pregnancies.**

Júlia A. Tronco^1^, Bruna R. de A. Ramos^1^, Natália M. Bastos^2^, Sérgio A. Alcântara^3^, Juliano C. da Silveira^2^, Márcia G. da Silva^1🖂^

^1^Department of Pathology, Botucatu Medical School, São Paulo State University (UNESP), Distrito de Rubião Júnior, Botucatu, São Paulo CEP 18618-686, Brazil

^2^Department of Veterinary Medicine, Faculty of Animal Science and Food Engineering, São Paulo University (USP), Pirassununga, São Paulo CEP 13635-900

^3^Department of Mophology, Biosciences Institute, São Paulo State University (UNESP), Distrito de Rubião Júnior, Botucatu, São Paulo CEP 18618-686, Brazil

**Supplementary Material**


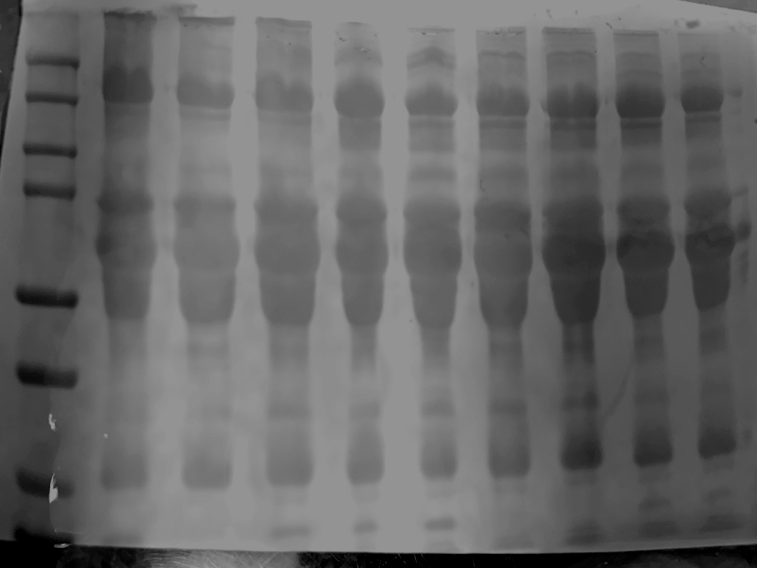


**Figure 1|** **Membrane stained with Ponceau used for normalization.** The Journal of Biological Chemistry^1^ and the American Journal of Physiology^2^ recently recommended Ponceau S, a total protein stain as the preferred method for normalization of Western Blots. According to the American Journal of Physiology, the assessment of loading using a total protein stain such as Ponceau S for membranes provides a better means than housekeeping protein analysis and allows normalization of each lane to the total protein in the lane.


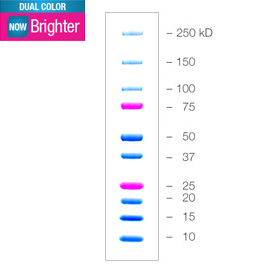


**Figure 2|** **Precision Plus Protein Dual Color Standards (Bio-Rad).** Protein ladder used for molecular weight estimation on western blot gels





**Figure 3|** Alpha-2-macroglobulin WB image





**Figure 4|** C1INH WB image

**

**

**Figure 5|** CD9 WB image





**Figure 6|** CD63 WB image





**Figure 7|** Hemopexin WB image





**Figure 8|** Cytochrome C WB image

**References**

1. Fosang, A.J., Colbran, R.J. Transparency Is the Key to Quality. *J Biol Chem*. **290**(50), 29692-4 (2015).

2. Brooks, H.L., Lindsey, M.L. Guidelines for authors and reviewers on antibody use in physiology studies. *Am J Physiol Heart Circ Physiol*. **314**(4), H724-H32 (2018).
